# Regular heartbeat rhythm at the heartbeat initiation stage is essential for normal cardiogenesis at low temperature

**DOI:** 10.1186/1471-213X-14-12

**Published:** 2014-02-25

**Authors:** Tomomi Watanabe-Asaka, Yoshio Sekiya, Hironori Wada, Takako Yasuda, Ikuya Okubo, Shoji Oda, Hiroshi Mitani

**Affiliations:** 1 Department of Integrated Biosciences, Graduate School of Frontier Sciences, The University of Tokyo, 5-1-5, Kashiwa-no-ha, Kashiwa, Chiba 277-8562, Japan; 2 PRESTO, Japan Science and Technology Agency (JST), Kawaguchi, Saitama 332-0012, Japan

**Keywords:** Heartbeat, Medaka, Blood regurgitation, Cold adaptation, Cardiogenesis

## Abstract

**Background:**

The development of blood flow in the heart is crucial for heart function and embryonic survival. Recent studies have revealed the importance of the extracellular matrix and the mechanical stress applied to the valve cushion that controls blood flow to the formation of the cardiac valve during embryogenesis. However, the events that trigger such valve formation and mechanical stress, and their temperature dependence have not been explained completely. Medaka (*Oryzias latipes*) inhabits a wide range of East Asia and adapts to a wide range of climates. We used medaka embryos from different genomic backgrounds and analyzed heartbeat characteristics including back-and-forth blood flow and bradyarrhythmia in embryos incubated at low temperature. We also used high-speed imaging analysis to examine the heartbeat of these animals after transient exposure to low temperature.

**Results:**

Embryos of the Hd-rR medaka strain exhibited back-and-forth blood flow in the heart (blood regurgitation) after incubation at 15°C. This regurgitation was induced by exposure to low temperature around the heartbeat initiation period and was related to abnormalities in the maintenance or pattern of contraction of the atrium or the atrioventricular canal. The Odate strain from the northern Japanese group exhibited normal blood flow after incubation at 15°C. High-speed time-lapse analysis of the heartbeat revealed that bradyarrhythmia occurred only in Hd-rR embryos incubated at 15°C. The coefficient of contraction, defined as the quotient of the length of the atrium at systole divided by its length at diastole, was not affected in either strain. The average heart rate after removing the effect of arrhythmia did not differ significantly between the two strains, suggesting that the mechanical stress of individual myocardial contractions and the total mechanical stress could be equivalent, regardless of the presence of arrhythmia or the heart rate. Test-cross experiments suggested that this circulation phenotype was caused by a single major genomic locus.

**Conclusions:**

These results suggest that cardiogenesis at low temperature requires a constant heartbeat. Abnormal contraction rhythms at the stage of heartbeat initiation may cause regurgitation at later stages. From the evolutionary viewpoint, strains that exhibit normal cardiogenesis during development at low temperature inhabit northern environments.

## Background

The heart is an organ that constantly circulates blood to the body, which is essential for animal survival. Congenital heart defects affect a few percent of human newborns each year [1,2]. Defects in the heartbeat are categorized into two types: impaired control of blood flow caused by a deformed valve and impaired contraction caused by abnormal cardiomyocytes. A rhythmic heartbeat is important for supplying oxygen to the whole body via the blood; therefore, arrhythmia is a relatively severe heart disease. In particular, the cardiac valve is essential to determining unidirectional blood flow, and 20–30% of cardiac defects are caused by heart valve abnormalities [2]. The recent literature has identified the key factors and pathways involved in valve development, such as extracellular matrix (ECM) formation and the importance of mechanical stress [3-6]. However, the mechanisms underlying the generation of the rhythmic contractions of the heartbeat during embryogenesis and the timing or events required for valve formation remain unknown.

Mutants with defects in heart formation have been isolated in medaka and zebrafish, some of which have heartbeat abnormalities [7-14]. The requirement of ECM components, such as chondroitin sulfate, for the formation of the atrioventricular canal (AVC) to prevent blood regurgitation during development of the heart has been shown in zebrafish embryos. The importance of mechanical stresses to valve formation was demonstrated by experiments that used microbeads to induce physical blockage and by mathematical models [4-7,9,15-17]. Temperature is one important factor that affects the growth of poikilothermic animals. Consequently, the acquisition of broad temperature adaptability enables expansion into a new environment. Temperature-sensitive mutants with abnormal phenotypes in heartbeat rhythm at the heartbeat initiation stage and blood flow regulation at the later stage, as detected using large-scale mutagenesis screening, have not been reported to date.

In this study, we identified the critical stage of normal heart development under low-temperature conditions using medaka embryos of an inbred strain derived from the “southern Japanese group” (S.JPN). High-speed image analysis revealed that the heartbeat rhythm at the stage of its onset was important for the establishment of normal blood circulation. Moreover, differences in heart development in embryos grown at cold temperature were observed between the S.JPN and the “northern Japanese group” (N.JPN). The genetic cross test used in this study showed that the cold-sensitive phenotype followed a Mendelian recessive inheritance.

## Results

### Blood regurgitation in medaka embryos grown at low temperature

**Figure 1:**
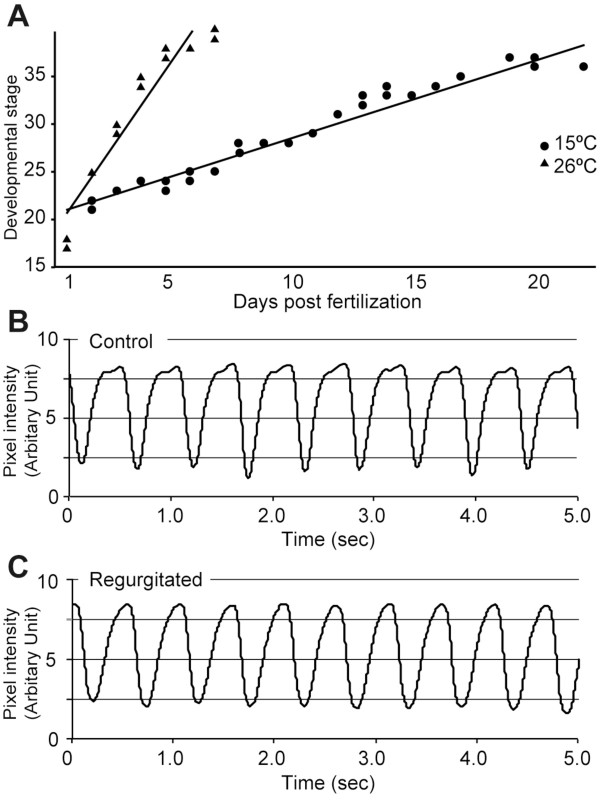
**Normal development and heartbeat analysis at st.34 in medaka embryos raised at 15°C. A**: Days and developmental stage for embryos incubated at 15°C and 26°C. Solid circles, embryos raised at 26°C; solid triangles, embryos raised at 15°C. **B** and **C**: Time course of the strength of the contraction of the heartbeat measured at 26°C in st.34 embryos (plotted for 5 s). **B**: control. **C**: regurgitation.

Before evaluating the influence on the heartbeat, the effect of low temperature on the total development was analyzed from the early neurula stage (developmental stage 17; st.17), which is after the body axis formation, in medaka embryos. Cold-treated (15°C) embryos of an S.JPN inbred strain (Hd-rR) showed normal development with slow but constant progress until they reached the hatching stage (st.39, about to hatch) (Figure 1A). Embryos at st.38, which is the spleen development stage, looked normal anatomically and exhibited normal neurological responses, such as eye movements and pectoral fin movements (data not shown). Axonal projections shown by immunohistochemical staining with an anti-acetylated α-tubulin antibody confirmed that their overall pattern, including the parasympathetic innervation to the heart, was unaffected (Additional file 1: Figure S1). However, their blood circulation was abnormal, with a back-and-forth blood flow (Additional file 2: Movies S1 and Additional file 3: Movies S2). Eighty-six percent (15 of 17) of the embryos that had been exposed to low temperature and that showed severe retrograde blood flow failed to hatch and exhibited severe edema in the head and heart region, and masses of blood cells in the heart and blood vessels around the hatching stage. Embryos that showed moderate regurgitation hatched at st.40. However, the larvae exhibited retrograde flow and could not swim because of degeneration of the pectoral and tail fins, possibly because of the lack of blood flow, and they died around 3 days after hatching. Several medaka mutants lacking heart functions also died either around the time of, or soon after, hatching [12,18].

To examine the regurgitation phenotype, we focused on st.34, the point at which blood circulation reaches the pectoral fin. Because it was conceivable that heart rate alterations are related to blood regurgitation, we examined the heart rate of the embryos at st.34. The heart rates of the embryos were measured at 26°C after incubation at 15°C or at 26°C (control). The average heart rate did not differ significantly between the control and cold-treated embryos exhibiting blood regurgitation: 116.0 ± 10.6 beats per minute (bpm) and 117.5 ± 1.82 bpm, respectively (Table 1, p > 0.46). The profiles of the heartbeat were also similar in the two types of embryos (Figure 1B and C). These results suggest that exposure to a low temperature affects the blood flow, although the heart itself retains a normal rate when analyzed at a normal temperature.

**Table 1:** Average heart rate of st.34 medaka embryos measured at 26°C

	**Hd-rR embryos**	
	**Control**	**Incubated at 15°C**
Heart rate (bpm; mean ± SD)	116.0 ± 10.6	117.5 ± 1.82

Average heart rate (bpm; measured at 26°C) at st.34 after incubation of the embryos at 15°C from st.18 onwards (“Incubated at 15°C”, n = 26) in the Hd-rR. All embryos incubated at 15°C showed blood regurgitation. Control embryos were incubated at 26°C and did not exhibit blood regurgitation (n = 27).

**Figure 2:**
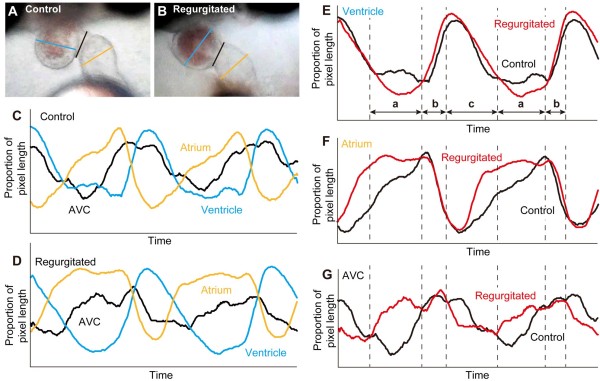
**Morphological characteristics of the ventricle, atrium, and AVC during a single heartbeat. A** and **B**: Examples of the heart at st.34 in the control **(A)** and regurgitating **(B)** medaka embryo. Light blue, orange, and black lines indicate the diameter of the ventricle, atrium, and AVC, respectively. **C** and **D**: External diameter of the ventricle, atrium, and AVC during a heartbeat in the control **(C)** and regurgitating **(D)** embryo. **E**–**G**: Characteristics of contraction of the ventricle **(E)**, atrium **(F)**, and AVC **(G)**. Red and black lines indicate the regurgitated and control embryos, respectively. a: The period when the ventricle maintains the contraction; b: The atrial contraction and ventricular dilatation period; c: the ventricular contraction period.

Next, to examine the possibility that an alteration in the contraction or expansion itself causes blood regurgitation, high-speed images were used to evaluate the patterns of contraction of the ventricle, atrium, and AVC at 26°C (Figure 2A–D). There were no gross morphological abnormalities in the three parts of the heart in the embryos exhibiting regurgitation (Figure 2A and B, and Additional file 4: Figure S2). The patterns of ventricular contraction and expansion were normal in the embryos exhibiting regurgitation (periods b and c in Figure 2E). However, the atrium expanded at the beginning of ventricular contraction in embryos exhibiting blood regurgitation (period c in Figure 2F). A similar pattern was observed in the AVC: the atrium started contraction (period b in Figure 2G), whereas the pattern was different during the atrium expansion (periods a and c in Figure 2F and G). These results suggest that the blood regurgitation observed in embryos grown at low temperature was related to abnormalities in the maintenance of contraction in the atrium and to the pattern of contraction in the AVC during normal contraction in the ventricle and atrium.

### Critical period for blood regurgitation at low temperature

**Figure 3:**
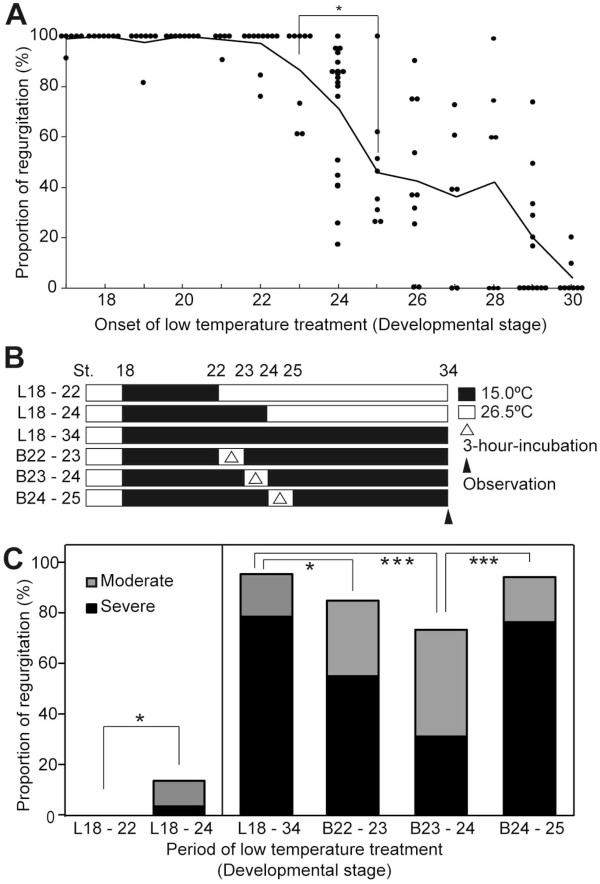
**Proportion of blood regurgitation phenotype after incubation at 15°C during embryogenesis. A**: Proportion of embryos showing regurgitation at st.34 after the onset of the 15°C treatment at the stages indicated (st.17, n = 50; st.18, n = 50; st.19, n = 54; st.20, n = 69; st.21, n = 53; st.22, n = 80; st.23, n = 66; st.24, n = 79; st.25, n = 65; st.26, n = 98; st.27, n = 50; st.28, n = 62; st.29, n = 86; st.30, n = 62). Only clutches with fertilized eggs (n ≥ 4) were used for the assay. Dots indicate each clutch. Lines indicate the average of proportions in clutches at the same stage. **B**: Scheme of the 15°C -treatment experiment used in C. Solid bar, 15°C; open bar, 26°C. L: Low-temperature treatment, B: Breakage of 15°C treatment. **C**: Comparison of the proportion of regurgitation between embryos at various stages (L18–22 (n = 189), L18–24 (n = 208), B22–23 (n = 40), B23–24 (n = 64), and B24–25 (n = 68)). Gray columns, moderate regurgitation; black columns, severe regurgitation. The classification of the regurgitation phenotype was performed by visual inspection. Asterisks indicated the level of significance (*: p < 0.05; ***: p < 0.005).

To identify the developmental stage that is critical for low-temperature-induced blood regurgitation, the Hd-rR strain was exposed to 15°C at different stages of development (Figure 3A). Blood regurgitation occurred in most embryos incubated at 15°C before the 12-somite stage which the tubular heart forms (st.23; no heartbeat) and decreased significantly when the incubation of embryos at 15°C began after the 18-19-somite stage which the circulation starts (st.25) (p < 0.05). Embryos did not exhibit regurgitation when exposed to cold after the 35-somite stage (st.30). Embryos incubated at 15°C from the late neurula stage (st.18) or the 16-somite stage when the first heartbeat appeared (st.24) to st.34 were raised at 26°C from st.34 to st.38. Twelve out of 12 embryos with low-temperature-induced blood regurgitation did not show clear recovery and 8 embryos failed to hatch. The rest of embryos died around 3 days after hatching. It is possible that some embryos incubated at 15°C from the 22-somite stage (st.26) or later could have recovered from regurgitation at a later time. To have induced retrograde blood flow after st.34 would have required some important event to occur between st.23 and st.25.

Subsequently, to identify the period that was most critical for blood regurgitation, Hd-rR embryos were incubated at 15°C from st.18 to the nine-somite stage (st.22) or to st.24, as depicted in Figure 3B. Blood regurgitation at st.34 did not occur in embryos exposed to cold at st.18–st.22 (low temperature: L18–22), although a percentage of the embryos showed blood regurgitation significantly increased to 12.1% (*P* = 0.014) in the embryos exposed to cold at st.18–st.24 (L18–24) (Figure 3C). In medaka, st.22, st.23 and st.24 embryos usually reach to the next developmental stages for 3, 3 and 6 hours at 26.5°C [19]. Next, the 15°C treatment was interrupted briefly by the 3-hour-incubation at 26.5°C from st.22 (breakage of 15°C treatment around st.22-23: B22–23), st.23 (B23–24) and st.24 (B24–25) to identify the stage that was critical for blood regurgitation. The frequency of the severe phenotype was significantly reduced in B22–23 and B23–24 embryos compare to the control embryos (Figure 2C; p = 0.019 and p = 0.0001, respectively). However, there was no rescue in the regurgitation phenotype by the break in the 15°C treatment at st.24–25. These results suggest that incubation at 15°C around the heartbeat initiation stage is sufficient to induce blood regurgitation in medaka and that this blood regurgitation phenotype is irreversible after st.34.

### Differences in phenotypic variations in blood regurgitation between medaka groups

**Figure 4:**
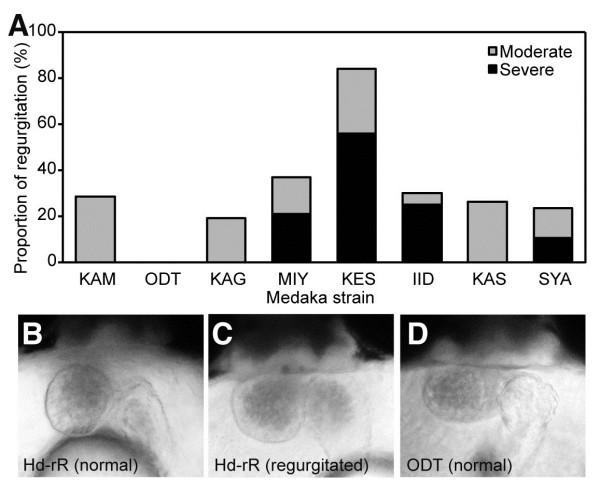
**Blood regurgitation at 15°C in medaka species and heart morphology at st.34. A**: Regurgitation ratio at st.34 after 15°C treatment from st.18 to st.34 in medaka strains from three medaka groups. The classification of the regurgitation phenotype was performed by visual inspection. KAM; n = 15, ODT; n = 38, KAG; n = 33, MIY; n = 23, KES; n = 37, IID; n = 46, KAS; n = 38, SYA; n = 53. **B**–**D**: Frontal view of medaka embryos at st.34. **B**: control Hd-rR. **C**: Hd-rR with regurgitation. **D**: ODT strain. KAM, Kamikita; ODT, Odate; KAG, Kaga; MIY, Mitatsu; KES, Kesennuma; IID, Iida; KAS, Kasumi; SYA, Shanghai.

To assess the relationship between resistance to low temperature and habitat in wild medaka populations, cold resistance was assessed by observing blood regurgitation, and this was compared between three groups of medaka: four strains of the N.JPN group; Odate (ODT), Kamikita (KAM), Kaga (KAG), and Miyatsu (MIY), three strains of the S.JPN group; Kesennuma (KES), Iida (IID), and Kasumi (KAS), and the Shanghai (SYA) strain, which was derived from the western Korean/Chinese group. Severe blood regurgitation without any other morphological abnormalities was observed in the KES and IID strains of the S.JPN group and in the SYA strain (Figure 4A). The KES strain of the S.JPN group exhibited the severe phenotype as seen in Hd-rR. The KAS strain exhibited mild regurgitation. Conversely, only moderate blood regurgitation was observed in the KAM and KAG strains of the N.JPN group, although the MIY strain exhibited severe regurgitation. The ODT strain was the most cold-resistant strain in terms of heart development in the N.JPN group (Figure 4A). Although the phenotype differed between the strains in each group, our results suggest that more medaka strains from the northern part of Japan than from the southern part of Japan tend to be tolerant to cold temperature-induced blood regurgitation during the breeding season.

### Comparative heartbeat analysis in st.24 and st.34 embryos of the Hd-rR and ODT strains

To examine cold sensitivity in the Hd-rR and ODT strains, we analyzed the heart rates of two strains at st.34. Heart morphology was the same in both strains at st.34 (Figure 4B–D). In st.34 ODT embryos, the heart rates were 67.3 ± 2.31 and 116.9 ± 6.44 bpm at 15°C and 26°C, respectively. In Hd-rR embryos, the heart rates were 70.7 ± 3.21 and 116.0 ± 10.6 bpm at 15°C and 26°C, respectively. The heart rates did not differ significantly between these two strains at either 15°C or 26°C (Table 2, p = 0.70).

**Table 2:** Average heartbeat measured at 15°C and 26°C in st.24 and st.34 medaka embryos of the Hd-rR and ODT strains

**Developmental stage**	**Heart rate (mean ± SD)**			
	**Hd-rR strain**		**ODT strain**	
	**15°C**	**26°C**	**15°C**	**26°C**
st.24	9.08 ± 1.66	24.0 ± 4.43	8.78 ± 2.67	21.1 ± 3.93
st.34	70.7 ± 3.21	116.0 ± 10.6*	67.3 ± 2.31	116.9 ± 6.44

Average heart rate (bpm) at st.24 and st.34 recorded 3 h after the onset of the 15°C treatment (15°C, n = 3 each) in the Hd-rR and ODT strains. Control embryos were incubated at 26°C (n = 3; n = 26 at st.34). The asterisk indicates the same data as presented in Table 1.

**Figure 5:**
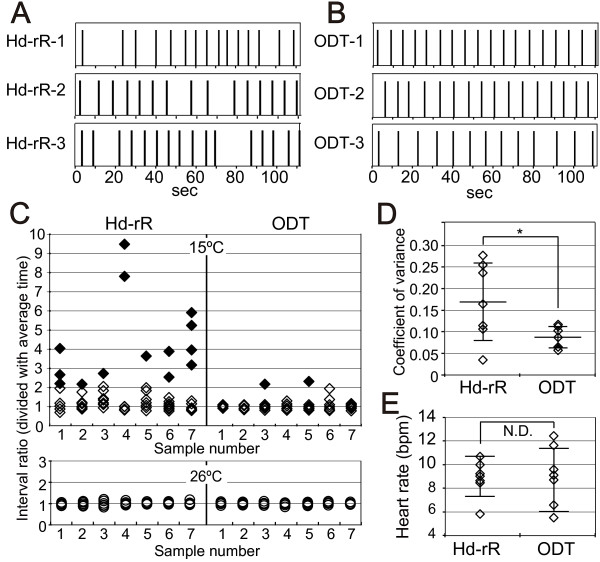
**Heart rate analysis at st.24. A** and **B**: The pacing of each heartbeat for 110 s in st.24 embryos incubated at 15°C. The beginning of contraction of the atrium is indicated by the bar. **A**: Hd-rR. **B**: ODT. **C**: Dispersion of heartbeat intervals. Ten heartbeats are shown for seven different embryos; the numbers 1–3 for Hd-rR and ODT refer to the numbers 1–3 in panels **A** and **B**, respectively. Solid diamonds show severe bradyarrhythmia, which was defined as an interbeat interval whose duration was more than twice that of the minimal interbeat interval. Each heartbeat was divided by the average heartbeat at 15°C and 26°C. Data for the Hd-rR and ODT strains are shown on the left and right, respectively. **D**: Coefficients of variance of heartbeats in seven embryos incubated at 15°C after eliminating bradyarrhythmia from the analysis are shown in solid diamonds in **C**. **E**: Average heart rates at 15°C, including prolonged heartbeats, among seven embryos of each strain in **C**. SDs are shown as error bars. Asterisks indicate the level of significance (*: p < 0.05).

To assess the impact of cold treatment on the heartbeat at st.24, we performed an imaging analysis of the heartbeat at 15°C in ODT and Hd-rR embryos at st.24. The rhythm of contraction at 15°C was almost constant in ODT embryos, whereas a severe delay in contraction occurred in Hd-rR embryos (Figure 5A and B). The variation in the interbeat interval was calculated by dividing the duration of each individual heartbeat by the average over 10 heartbeats and was examined in st.24 embryos of the Hd-rR and ODT strains (Figure 5C). Bradyarrhythmia was defined as an interbeat interval whose duration was more than twice the minimal interbeat interval. Most of the Hd-rR embryos incubated at 15°C showed bradyarrhythmia (shown in solid diamonds in Figure 5C). Bradyarrhythmias were then excluded from the following analysis. The coefficient of variance was determined as the quotient of the variance divided by the average interbeat interval. The coefficient of variance was significantly larger in the Hd-rR strain: 0.09 ± 0.025 and 0.17 ± 0.09 in ODT and Hd-rR embryos, respectively (p = 0.035) (Figure 5D). Conversely, no difference in the coefficient of variance was observed in embryos incubated at 26°C (Figure 5C).

To verify the possibility that a reduction in heart rate caused blood regurgitation, the average heart rate was calculated for 10 beats. Some ODT embryos exhibited a slower average heart rate than did Hd-rR embryos (Figure 5E). Average heart rate including arrhythmias in the Hd-rR and ODT embryos are 8.48 ± 2.74 and 5.74 ± 1.51, respectively (p <0.05). This result apparently seems that heart rate under low temperature is slower in the Hd-rR strain than the ODT. However the difference between these strains is mainly due to the bradyarrhythmia in the Hd-rR strain. After removing the bradyarrhythmias to compare the intrinsic heart rate under low temperature, the average heart rates did not differ between the Hd-rR and ODT strains: 9.08 ± 1.66 and 8.78 ± 2.67 bpm, respectively (Figure 5E and Table 2; p = 0.80). The heart rate was constant, and no arrhythmia was observed in either strain at 26°C (Table 2). These results suggest that incubation at 15°C during the early stage of development of the heartbeat disturbs the rhythm but not the heart rate in Hd-rR embryos.

Terfenadine is a K^+^-channel blocker that disturbs the heartbeat even in small teleosts [20,21]. To examine whether K^+^-channel blockage could account for the observed rhythm phenotype of the heartbeat, terfenadine was added to Hd-rR and ODT embryos at st.24 and st.34. Atrioventricular blockage was induced in the terfenadine-treated embryos at the heart development stage (st.36) without blood regurgitation. By contrast, terfenadine did not affect the heartbeat at st.24 (data not shown).

**Figure 6:**
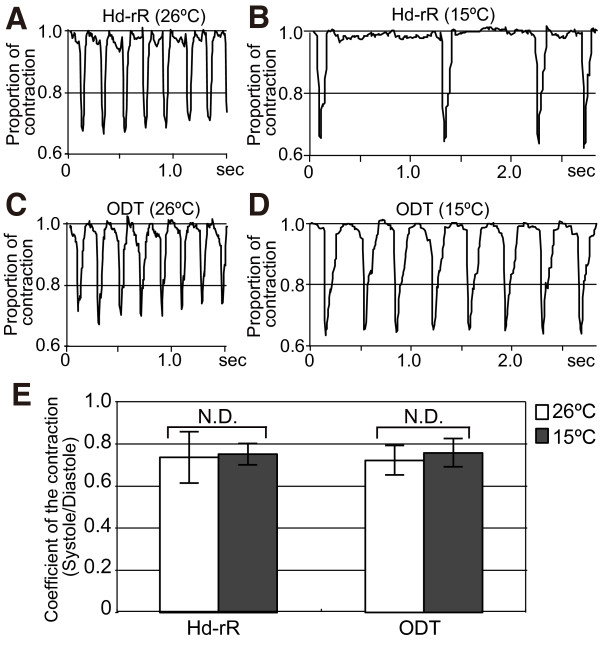
**Analysis of the heart contraction at st.24. A**–**D**: Time-course analysis of the heartbeat at 26°C or 15°C in st.24 embryos. **A** and **C**: st.24 embryos at 26°C, plotted for 1.5 s. B and D: st.24 embryos at 15°C, plotted for 2.8 s. **A** and **B**: Hd-rR. **C** and **D**: ODT. **E**: Averages of the coefficients of contraction for five typical heartbeats in three embryos of ODT and Hd-rR. Open columns, 26°C; solid columns, 15°C. The error bars denote the SDs.

To examine the quality of heartbeats in the Hd-rR and ODT strains at st.24, the pattern of contraction was plotted (Figure 6A–D). The time to contraction and the duration of the contraction did not differ between strains or temperature. We defined the coefficient of contraction as the quotient of the length of the atrium at systole divided by its length at diastole. The coefficient of contraction was around 0.7 in both Hd-rR and ODT embryos, and this coefficient did not differ between ODT and Hd-rR embryos incubated at either 15°C or 26°C (Figure 6E). These results suggest that the mechanical stress of each individual myocardial contraction was equivalent, regardless of the presence of arrhythmia or the heart rate.

### Pattern of inheritance of the blood regurgitation and bradyarrhythmia phenotype

**Figure 7:**
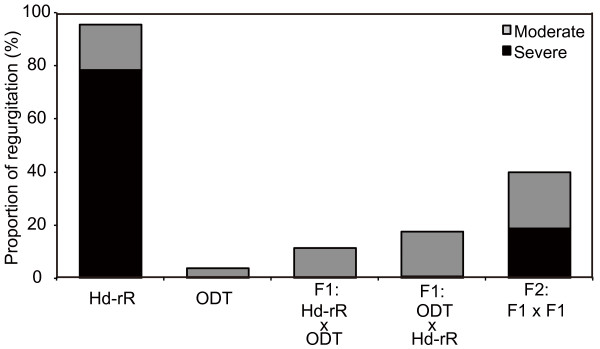
**Blood regurgitation at 15°C in medaka species.** Genetic analysis in the Hd-rR (n = 210) and ODT (n = 205) strains. F1 were generated both via the Hd-rR female × ODT male combination (n = 215) and ODT female × Hd-rR male combination (n = 206). F2 (n = 213) were generated by intercrossing F1 progeny. Gray columns, moderate regurgitation; black columns, severe regurgitation.

Finally, to examine the possibility that the regurgitation was caused by the genetic background, blood regurgitation caused by low temperature was examined in the F1 and F2 progeny of the intercross between the Hd-rR and ODT strains (Figure 7). The frequency of embryos with regurgitation in the Hd-rR and ODT parent generation was about 95% and 5%, respectively. In the F1 generation, regardless of the maternal background of the embryo, the incidence of regurgitation was 14.6% on average, and the incidence of the severe phenotype was 0.65% (Figure 7). The incidence of regurgitation in the F2 generation was 39.9%, whereas that of the severe phenotype was 18.9% (Figure 7). The frequency of bradyarrhythmia at 15°C was also examined in the F2 offspring of which outcross-intercross and outcross-backcross between the Hd-rR and ODT strains at st.24 (Table 3). Twenty-one and fifty-four percent of embryos showed bradyarrhythmia in the outcross-intercross and outcross-backcross F2 offspring, respectively. These results suggest that in the F1 generation, the Hd-rR trait of cold-elicited severe blood regurgitation was recessive to normal rhythms observed in ODT embryos.

**Table 3:** Frequency of braddyarrhythmia at 15°C in st.24 and st.34 medaka embryos of the Hd-rR and ODT strains

	**Total**	**Phenotype (%)**	
		**Arrhythmia**	**Normal**
F1 × F1	28	6 (21.4)	22 (78.6)
F1 × Hd-rR	113	61 (54.0)	52 (46.0)

The heartbeat was examined at st.24 recorded 3 h after the onset of the 15°C treatment in the F1 intercross (F1 × F1) and F1 backcross (F1 × Hd-rR) embryos.

## Discussion

In this study, we showed that a blood regurgitation phenotype resulting from incubation at 15°C was present in an inbred medaka strain derived from the S.JPN group. Moreover, we found that a constant heartbeat at low temperature could be an important factor for normal blood circulation. Embryos with blood regurgitation at st.34 exhibited a constant heartbeat without neural abnormalities; however, the circulation phenotype observed in this study was irreversible and may have been caused by abnormalities in the maintenance of the contraction in the atrium and the pattern of the contraction in the atrioventricular canals. Hence, these results suggest the importance of the heartbeat rhythm at the heartbeat initiation stage and that this phenotype arises from a single genetic locus.

(a) Mechanisms causing the irregular heartbeat observed at low temperature

Our results showed that low temperature caused bradyarrhythmia in Hd-rR embryos at the heartbeat initiation stage. In the chick embryo, this heartbeat initiation stage is highly sensitive to low temperature, and cooling causes disorder in the rhythmicity of the action potential [22]. During embryogenesis, the heart is the only organ that has rhythmicity; therefore, the blood circulation would be especially sensitive to low temperature. It is thought that the Ca^2+^ channel functions as the pacemaker in the early embryonic heart and that it is important to maintain the Ca^2+^ signal in the cytoplasm [23]. The results of the present study show that the K^+^ channel is irrelevant, implying that the stability of Ca^2+^ channels might be crucial at low temperatures. It is known that the rhythmicity of this Ca^2+^ concentration is mediated by several components such as hyperpolarization-activated cation channel 4; however, the interactions of these molecules are complicated.

The ODT strain is derived from the N.JPN group and has a distant genetic background from that of the Hd-rR strain. The ODT strain has tolerance to the regurgitation phenotype under low temperature. In this study, intercrossing between the Hd-rR and ODT strains showed that the blood regurgitation phenotype followed a Mendelian inheritance pattern. It has been shown that mitochondrial function differs between medaka local groups at low temperature [24]; however, the similar phenotype observed in reciprocal F1 hybrids suggests that polymorphisms in the mitochondrial genome do not affect the induction of blood regurgitation by cold temperature. The results of the test-cross experiments suggest that the blood regurgitation observed in the Hd-rR strain at low temperature is controlled by a single major locus. The whole-genome sequence of the Hd-rR has been decoded, and backcrossing with the ODT strain followed by phenotype selection should lead to the identification of the genetic region involved in blood regurgitation.

(b) Adaptation to low temperature in medaka wild populations

In their natural environment, medaka in Japan spawn from the end of April to August. It is known that embryos of medaka strains of the S.JPN group develop normally at 16–34°C and that blood stagnation or impaired blood vessel formation causes failure in hatching at temperatures below 16°C [25]. However, at the beginning of spring, medaka embryos in the northern part of Japan frequently experience temporarily low temperatures below 16°C in the field [25-27]. Odate, which is located at high latitude, is a habitat of the N.JPN population and is the original location for the capture of fish of the ODT strain. The maximum and minimum temperatures in May (spawning period) are around 20°C and 10°C, respectively (http://www.data.jma.go.jp/obd/stats/etrn). About 100 wild populations of medaka have been maintained as closed colonies in an outdoor breeding farm at the University of Tokyo, Graduate School of Frontier Sciences, since 1985 [28]. The maximum and minimum water temperatures at which the N.JPN group, including the ODT strain, starts spawning are around 18°C and 10°C, respectively.

The MIY strain of the N.JPN group and the KAS strain of the S.JPN group are distributed at the border between the N.JPN and S.JPN groups, which is at a lower latitude than that of the ODT strain habitat. The SYA strain of the western Korean/Chinese group showed blood regurgitation, suggesting that *Oryzias latipes* acquired resistance to a cold environment after reaching Japan. The genetic diversity of the medaka species enables its distribution across habitats with various environments, and the responsive gene for cold resistant heart development should be one of the master genes for the expansion of this species into cold habitats.

(c) Relationship between the disturbance of the heart rhythm and cardiac hypoplasia

The results in this study suggest that the regurgitation observed after incubation at low temperature was caused by a structural defect, such as in the function of the canals or the atrium, and not by a transient, morphological or neurogenic dysfunction. Three possible mechanisms may have caused the blood regurgitation phenotype observed: an unclosed valve that was not formed properly, an irregular rhythm, or abnormal atrial and ventricular contractions. Our results showed that the rhythm of the heartbeat and the coefficient of the contraction were not affected in the regurgitated embryos at st.34. There were no apparent morphological abnormalities; therefore, very subtle differences in the AVC structure may be important to continue proper heartbeat. Conversely, the presence of retrograde flow with or without valves in some zebrafish mutants has been reported [7,9]. The endocardial cushion plays a role as a valve in the early stages of zebrafish and medaka development [12,29,30]. Cardiac physiologists have long conjectured that the valveless embryonic heart tube drives circulation via peristaltic or impedance contractions [5,31-33].

It has been reported that the heart formation of medaka embryo of st.34 is similar to zebrafish embryos of stage 56 hours post fertilization (hpf) [12] and that the cardiac valve at 56 hpf is known as the cushion in zebrafish [4]. The heartbeat could be distinguished in the period of the isovolumic ventricular contraction period (a in Figure 2E–G), the atrial contraction/ventricular dilatation period (b in Figure 2E–G), and the ventricular contraction and ejection period (c in Figure 2E–G). The results of this study show normal contractions in the ventricle (b and c in Figure 2E–G) and in the atrium (b in Figure 2E–G), and abnormalities in the maintenance of the contraction in the atrium and pattern of the contraction in the AVC. The diameter of the ventricle during period a in Figure 2E–G is smaller in the regurgitating embryo. It is possible that the ventricular diameter is reduced by the increased pressure in the pericardium caused by the relaxed atrium and AVC. This might explain the blood regurgitation observed in the Hd-rR strain.

The heart in medaka is positioned between the yolk and the embryo during embryogenesis, and this situation causes difficulties observing the outflow tract in medaka. However, it is less likely that the blood regurgitation is caused by the structure of the outflow tract because of the normal ventricular contraction in the period (c in Figure 2E–G). Hence, the low-temperature-induced regurgitation may reflect an abnormality in the pattern or in the maintenance of the contraction in the atrium or the AVC, especially in the endocardial cushion. The results showing that a constant heartbeat was necessary for normal development in the ODT strain at 15°C in the present study support the importance of sheer stress induced by the rhythmic contraction to the retrograde flow afterwards. Unidirectional rhythmic contraction is required for ATP release and Ca^2+^ signaling in blood vessels and microRNA expression in the valve appear with the heartbeat [34,35]. A similar mechanism could also play an important role in the initial stages of heart formation. Direct evidence about whether a structural defect exists is needed to confirm this hypothesis in the future.

## Conclusions

In conclusion, the low-temperature-induced disturbance of the rhythm of the heartbeat in these embryos was associated with irreversible blood regurgitation observed in the AVC during embryonic development at later stages. Intercrossing experiments suggested the existence of a single major gene related to this phenotype. The identification of the genetic locus that contributes to cold sensitivity in medaka would represent another breakthrough in the study of cold adaptation and of its relationship with the population diversity of this species.

## Methods

### Fish maintenance and husbandry

The medaka (*Oryzias latipes*) inbred strain Hd-rR (derived from the S.JPN group) was obtained from our breeding colony. We also used local strains (demes): ODT, KAM, KAG, and MIY strains from the N.JPN group; KES, IID, and KAS strains from the S.JPN group; and SYA strain from a western Korean/Chinese group. All animals in this study were kept as closed colonies under laboratory conditions. Eggs were collected and incubated in Petri dishes (φ 60 mm) with tap water containing 0.0001% methylene blue. The designation of medaka developmental stages was in accordance with that of Iwamatsu (2004) [19]. The fish were maintained under standard laboratory conditions at 26–29°C with a 14 h:10 h light:dark photoperiod and were fed three times a day with live brine shrimp and commercial pellet food. No significant differences in whole-body development were observed between strains. The Committees for Institutional Animal Care of the University of Tokyo approved the animal protocols.

### Low-temperature treatment and observation of the blood circulation

The fertilized eggs were incubated at ambient temperature (~26.5°C) until the developmental stage needed for the experiments. In each experimental condition, more than 30 embryos were exposed to a temperature of 15°C at certain periods of development. The egg water was changed once a week. The contraction of the heart at st.24 was calculated after a 4 h incubation at 15°C. In the control medaka, there was some retrograde blood flow until st.33, the stage at which notochord vacuoliazation is completed, and chorions became turbid and fragile at st.39 before hatching. Heartbeat and blood circulation were observed at st.24, st.34, and st.38. Blood flow was classified into three patterns based on the level of the back flow to the atrium: the absence of back flow or the return of a few dozen blood cells into the atrium was classified as “normal”; the presence of blood regurgitation but with continuing circulation was classified as “moderate”; and the cessation of circulation because of blood regurgitation into the heart was classified as “severe”.

### Digital video recording, extraction of images showing heart movement, and heartbeat analysis

Embryos in the chorion were immobilized in the correct orientation using 2.5% methylcellulose in glass-bottom dishes. Terfenadine (final concentration 10 μM in 0.1% DMSO) was added 2 h before video recording. Retrograde blood flow from the ventricle to the atrium was assessed at 15°C or 26°C using a stereomicroscope (Nikon MULTIZOOM AZ100) equipped with a digital high-speed camera (CASIO Exilim EX-F1) [36]. Digital pictures were captured at 30 or 300 frames/s (fps) at a resolution of 512 × 384 pixels for up to 3 min and recorded in a PC using Final Cut Pro software (Apple Inc., Cupertino, USA).

Movies of heart movements were processed using Bohboh software (BohbohSoft, Tokyo, Japan). Blood flow and contraction rhythms were measured based on alterations in the intensity of blood flow into and out of the atrium. Regions of interest (ROIs) in the atrium were selected. The pixel intensities of the ROIs were digitized throughout the entire time series examined using Bohboh and were processed further using Cutwin mathematical software (EverGreen Soft, Tokyo, Japan). The data were processed using a smoothing algorithm by taking the movement average over 21 frames to correct for peak-detection errors. Average heart rates were obtained from the number of maxima recorded in 1 min. The time between maxima gave a measure of the interbeat interval. Bradyarrhythmia was identified as the interbeat interval whose duration was more than twice that of the minimal interbeat interval. Bradyarrhythmias were excluded from the coefficient of variance and heart rate analyses. Heart rate variation was obtained as the difference of each interbeat interval from the average value.

The external diameter of the ventricle, atrium, and AVC were selected. The length of each region was digitized throughout the entire time series examined using Bohboh and processed further using Cutwin. The data were processed using a smoothing algorithm by taking the movement average over 21 frames to correct for peak-detection errors. Two heartbeats were plotted as typical heartbeats. The coefficient of the contraction was calculated as the quotient of the length of the atrium at systole divided by its length at diastole. Calculations were performed three times in each embryo.

### Statistical analyses

Statistical analyses were performed using Fisher’s exact test and one-way ANOVA using JMP software (SAS Japan, Tokyo, Japan). A p value < 0.05 was considered significant. Data are presented as the mean ± SD of the indicated samples per experiment.

## Abbreviations

AVC: Atrioventricular canal; ECM: Extracellular matrix; fps: Frames per second; IID: Iida; KAG: Kaga; KAM: Kamikita; KAS: Kasumi; KES: Kesennuma; MIY: Mitatsu; N.JPN: The Northern Japanese group; ODT: Odate; ROI: Region of interest; SK2: Sukesuke; S.JPN: The Southern Japanese group; SYA: Shanghai.

## Competing interests

The authors declare that they have no competing interests.

## Authors’ contributions

TW-A performed the imaging analysis and drafted the manuscript. YS performed the phenotype screening and statistical analysis, and drafted the manuscript. HW performed the immunostaining. TY performed the histological analysis. IO participated in the test-cross experiment. SO participated in the design of the study and helped draft the manuscript. HM conceived the study, participated in its design and coordination, and helped draft the manuscript. All authors read and approved the final manuscript.

## Supplementary Material

Additional file 1: Figure S1 The overall patterning of the cranial nerves and their innervation to the heart were unaffected in low-temperature-treated embryos. (A, B) Low-temperature-treated embryos were fixed at st.34 and stained with an anti-acetylated α-tubulin antibody. Lateral (A) and ventral (B) views of the head region under a dissecting fluorescence microscope. The arrows indicate the caudal-most peripheral branch of the vagus nerve. (C, C′) High-magnification images taken under a confocal microscope. Lateral views. A fine branch (arrows) bifurcating from the main vagus nerve (arrows) was detected. (D) Same sample, ventral view. The fine branch (arrows) extended only from the left vagus nerve and crossed the midline to innervate the heart (indicated by a dotted line). The axonal projections to the heart were not altered in low-temperature-treated embryos compared with wild-type control embryos (data not shown). Immunohistochemistry was performed according to standard protocols [37] using an anti-acetylated α-tubulin antibody (Sigma-Aldrich Corporation, St. Louis, USA; dilution, 1:1000) and an anti-rabbit IgG conjugated to Alexa Fluor 488 (Molecular Probes, Life Technologies Corporation, Carlsbad, USA; 1:500). Click here for file

Additional file 2: Movie S1 Frontal view of an Hd-rR control embryo at st.34 (300 fps movie). The blood flow was unaffected in low-temperature-treated embryos. Click here for file

Additional file 3: Movie S2 Frontal view of an Hd-rR embryo with regurgitation at st.36 (300 fps movie). Blood flow was regurgitated in the heart. Click here for file

Additional file 4: Figure S2 The overall patterning of the heart at st.36 was unaffected in low-temperature-treated embryos. (A and B): Schematic diagram of a lateral view of the medaka embryo and the heart at st.36, illustrating where the section was made (black line). (C and D): Transverse sections of the heart at st.36 in the control (C) and regurgitating (D) embryos. Eight-micrometer thick sections were prepared and stained with hematoxylin and eosin [38]. a: atrium, b: bulbus arteriosus, v: ventricle. Click here for file
