# Supplementary figures and images for: Regular heartbeat rhythm at the heartbeat initiation stage is essential for normal cardiogenesis at low temperature
Source: BMC Dev Biol. 2014 Feb 25;14:12. doi: 10.1186/1471-213X-14-12 (PMC3936829; doi:10.1186/1471-213X-14-12)

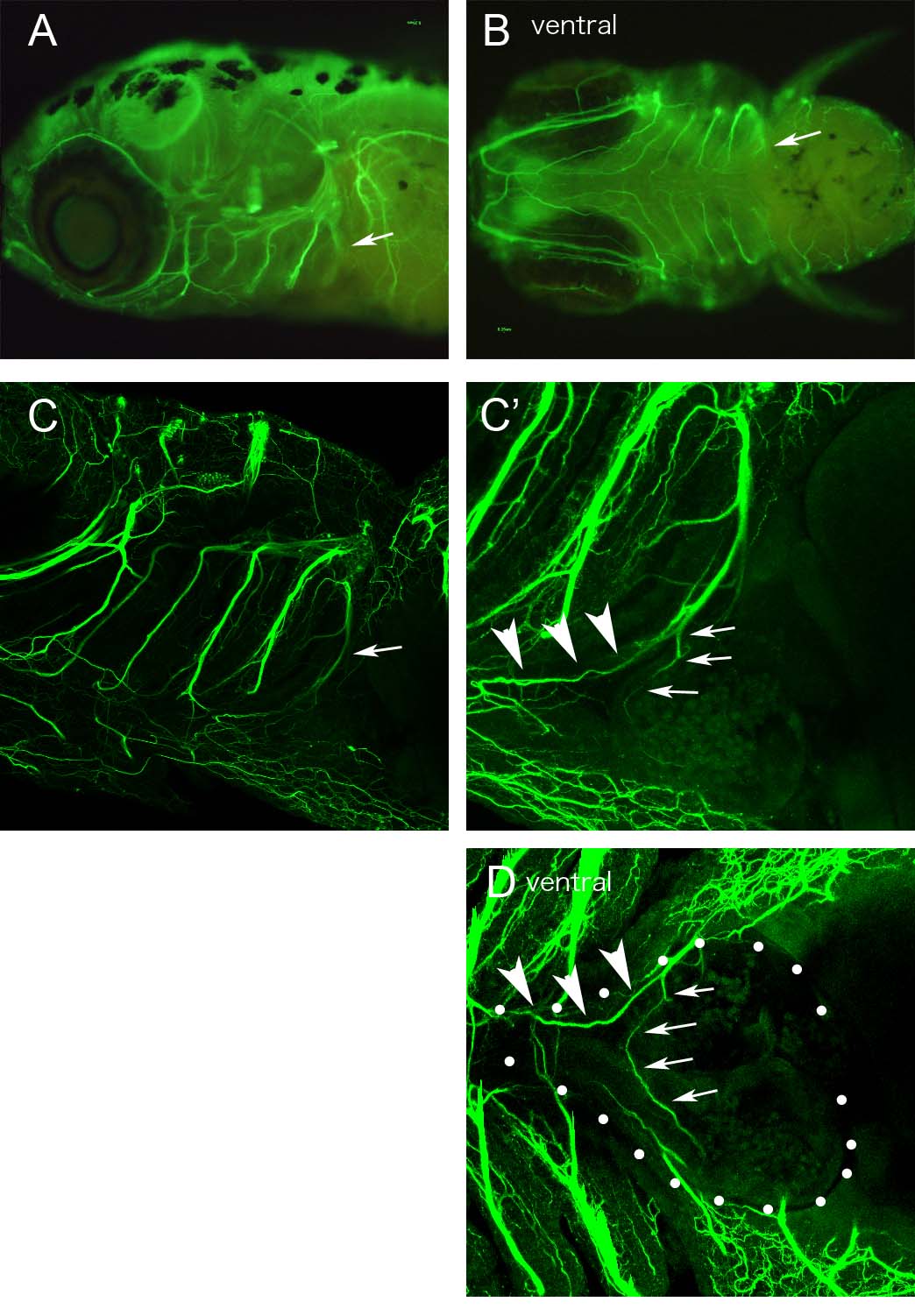

Supplement: Additional file 1: Figure S1 — The overall patterning of the cranial nerves and their innervation to the heart were unaffected in low-temperature-treated embryos. (A, B) Low-temperature-treated embryos were fixed at st.34 and stained with an anti-acetylated α-tubulin antibody. Lateral (A) and ventral (B) views of the head region under a dissecting fluorescence microscope. The arrows indicate the caudal-most peripheral branch of the vagus nerve. (C, C′) High-magnification images taken under a confocal microscope. Lateral views. A fine branch (arrows) bifurcating from the main vagus nerve (arrows) was detected. (D) Same sample, ventral view. The fine branch (arrows) extended only from the left vagus nerve and crossed the midline to innervate the heart (indicated by a dotted line). The axonal projections to the heart were not altered in low-temperature-treated embryos compared with wild-type control embryos (data not shown). Immunohistochemistry was performed according to standard protocols [37] using an anti-acetylated α-tubulin antibody (Sigma-Aldrich Corporation, St. Louis, USA; dilution, 1:1000) and an anti-rabbit IgG conjugated to Alexa Fluor 488 (Molecular Probes, Life Technologies Corporation, Carlsbad, USA; 1:500). [file 1471-213X-14-12-S1.jpeg]

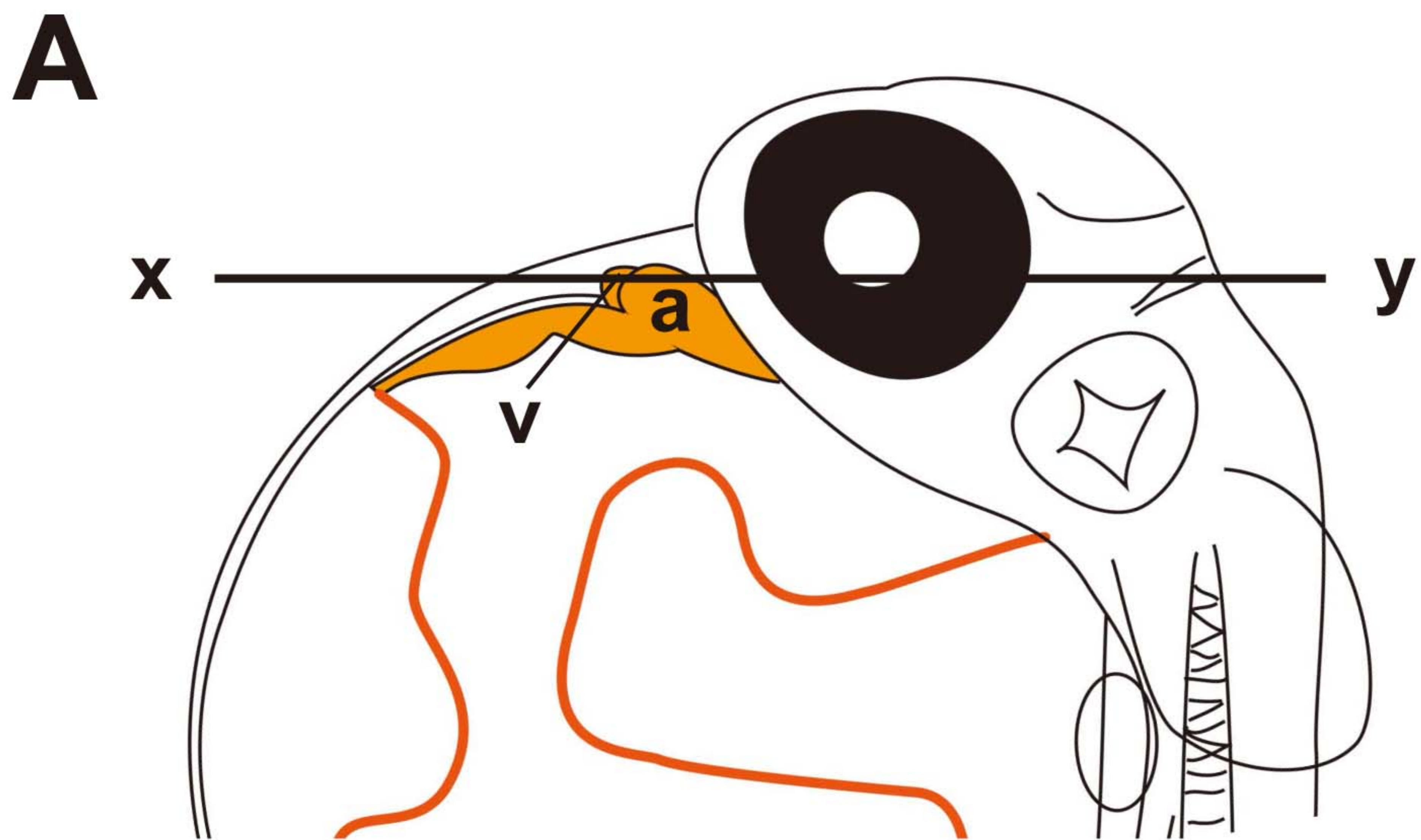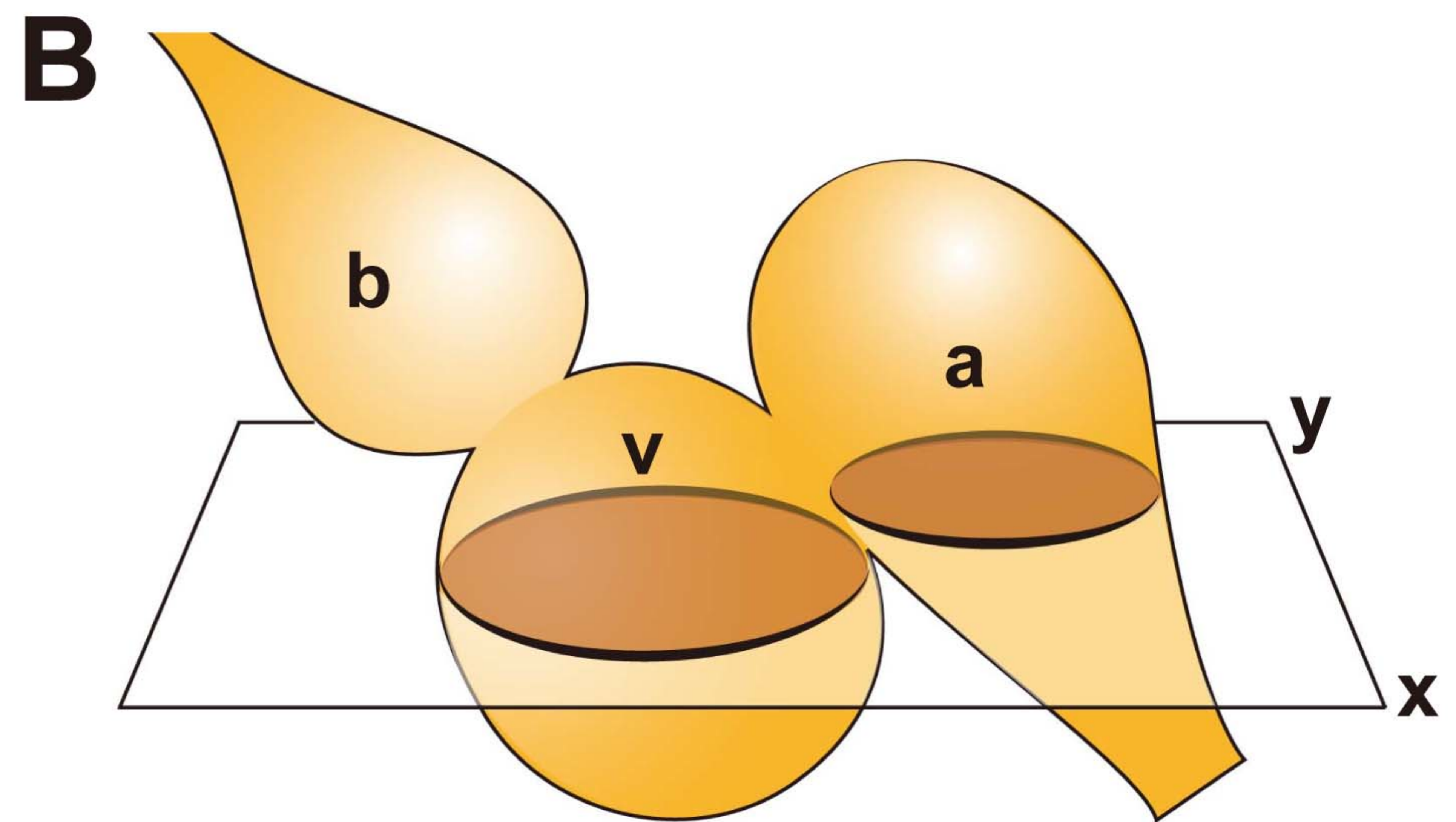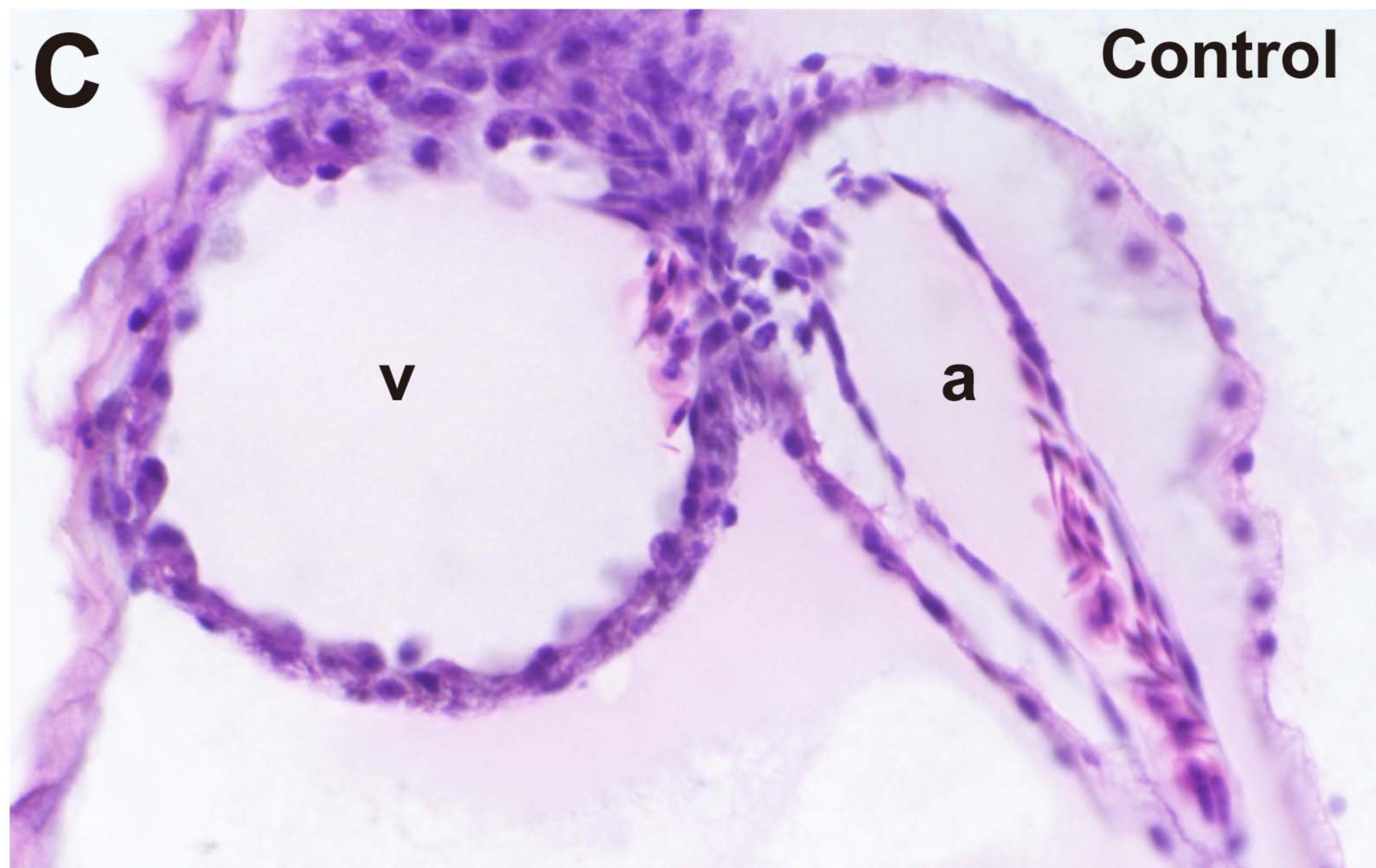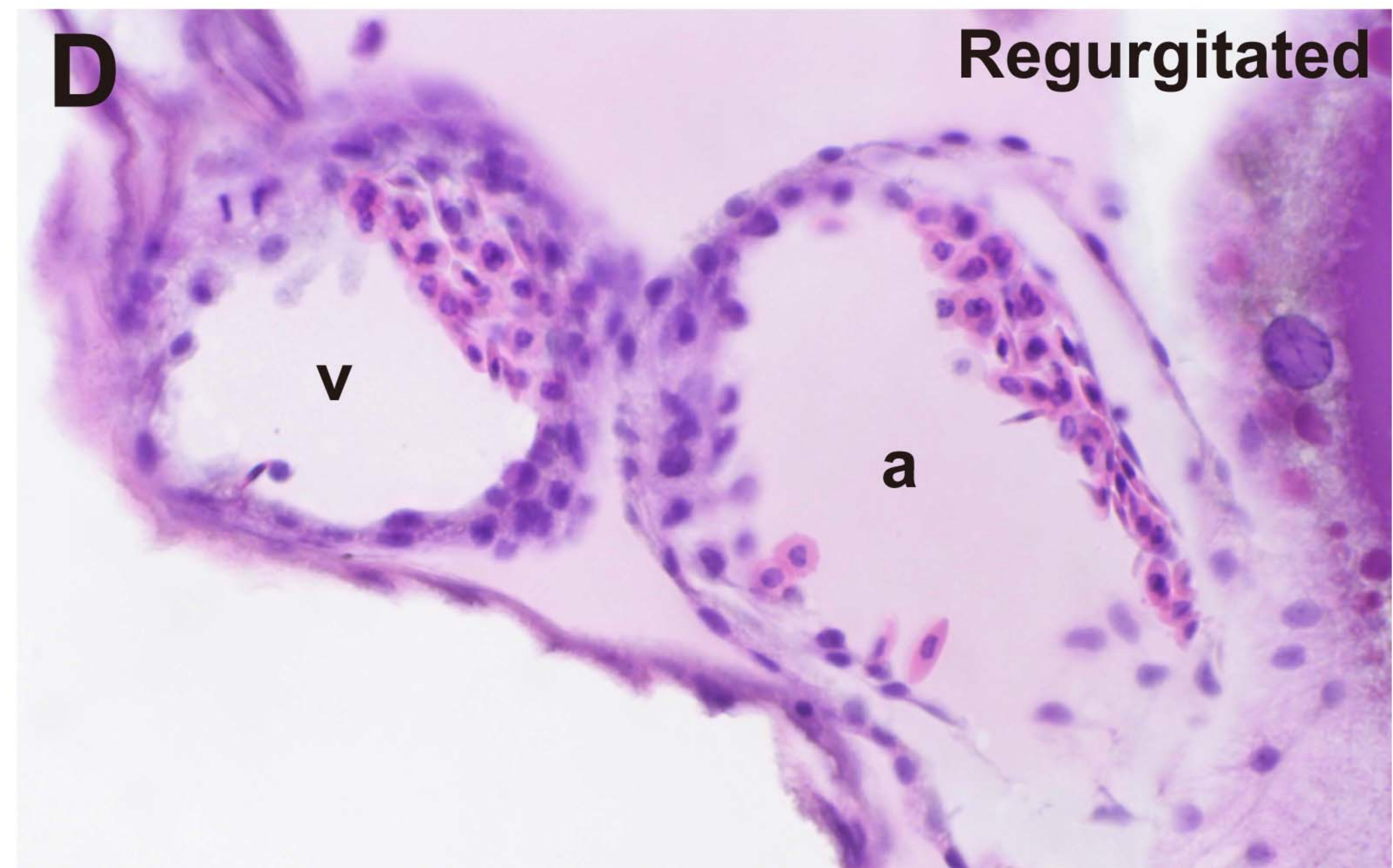

Supplement: Additional file 4: Figure S2 — The overall patterning of the heart at st.36 was unaffected in low-temperature-treated embryos. (A and B): Schematic diagram of a lateral view of the medaka embryo and the heart at st.36, illustrating where the section was made (black line). (C and D): Transverse sections of the heart at st.36 in the control (C) and regurgitating (D) embryos. Eight-micrometer thick sections were prepared and stained with hematoxylin and eosin [38]. a: atrium, b: bulbus arteriosus, v: ventricle. [file 1471-213X-14-12-S4.pdf]
